# Obstructive Sleep Apnea Syndrome in women: gender in sleep respiratory medicine is a first step towards personalized medicine

**DOI:** 10.1007/s11325-025-03420-1

**Published:** 2025-07-23

**Authors:** Caterina Antonaglia, Gloria Maria Citton, Matteo Siciliano, Barbara Ruaro, Francesco Salton, Marco Confalonieri

**Affiliations:** 1 Department of Medical Surgical and Health Sciences, Pulmonology Unit, Hospital of Cattinara, University of Trieste, 34149 Trieste, Italy; 2 Fondazione Policlinico Universitario A. Gemelli IRCCS- Università Cattolica del Sacro Cuore, Rome, Italy

**Keywords:** Gender medicine, Women with OSA, Sleep respiratory disordered in women, Gender differences in sleep medicine, Obstructive sleep apnea

## Abstract

Obstructive sleep apnea syndrome is the most common sleep disordered breathing. In recent years, literature has focused on the anatomical and functional factors in the pathophysiology of patients to better understand their clinical and polysomnographic features, aiming for personalized treatment. Studies suggest that sleep-disorders breathing in women are underestimated and associated with cardiovascular and metabolic disorders. Women tend to experience more mood disorders and behavioral symptoms, leading to a reduced quality of life. The sleep and respiratory pathophysiology in women are complex and varies with age and hormonal status. This review summarizes recent knowledge on this topic, emphasizing the pathophysiological mechanisms to explain the typical clinical and polysomnographic patterns observed in females. In sleep respiratory medicine, gender- specific approaches are now essential for personalized patient care.

## Introduction

Obstructive Sleep Apnea Syndrome (OSAS) is the most common sleep disordered breathing (SDB) and the most common sleep disorder in general after insomnia. SDB, encompassing OSAS and other abnormal disorder breathing during sleep (including snoring, upper airway resistance and central sleep apnea), has been estimated to have a male to female ratio between 3:1 and 5:1 in the general population [1, 2].

In the last few years an increasingly large body of evidence suggested that women are also significantly affected by OSAS, which is associated with cardiovascular and metabolic disorders similarly to men. However, the symptoms are often different: female patients with OSAS experience more mood disorders or behavioral symptoms than males, resulting in a higher reduction in quality of life [3, 4]. Indeed, instead of the prominent snoring and witnessed apneas often seen in male patients, female patients may experience subtler symptoms such as insomnia, headaches, fatigue, and mood disturbances. This difference in symptomatology can contribute to underdiagnosis and misdiagnosis, delaying a timely treatment.

The prevalence of OSAS in women, along with its clinical and polysomnographic aspects, varies across different life stages: pregnancy, pre- and post-menopause, and aging, leaving room to hypothesize that hormonal changes play a role [5]. The pathophysiology of OSAS is complex and is further complicated by gender differences. Interest in gender medicine is growing, prompting a focus on the pathophysiology of OSAS in women. Understanding these differences is crucial for improving diagnostic and treatment approaches tailored to female patients. This entails a comprehensive approach that considers the physiological, hormonal, and psychosocial factors unique to women, which are covered by this review.

### Sleep and hormonal changes in women

The female sex is characterized by a greater quantity of sleep but poorer sleep quality compared to males. Women, in particular, experience longer sleep latency despite an increase in slow-wave sleep time relative to men. Nevertheless, they are more prone to insomnia [6]. They also differ in their chronotype: women are larks (wake up early in the morning), suffer less from jet lag, but have an earlier sleep cycle than men. In summary women have more sleep disorders in general while men are much more prone to sleep apnea [7]. Sex hormones influence both sleep and the respiratory system [8]. In women, hormonal changes over the course of a lifetime provide protection especially against respiratory sleep disorders in the fertile age, which is lost in the menopausal age. Another aspect that is often underestimated is the impact of monthly hormonal fluctuations. Hormones affect sleep, and the premenstrual drop in estrogen and progesterone can, in some women, lead to sleep disorders as part of another condition: premenstrual dysphoric disorder [9]. Little is known about the role of hormonal contraceptives on sleep and sleep disorders in general, and although quality and efficiency of sleep seem to be better with these drugs, more research is needed [10]. Regarding respiratory sleep disorders, carbon dioxide sensitivity is higher and upper airway resistance is lower during the luteal phase (when progesterone levels are high) compared to the follicular phase. Therefore, the day of the menstrual cycle chosen to perform a home sleep test is not an insignificant detail [8]. Hormonal dysfunctions, such as polycystic ovarian syndrome (PCOS), also influence sleep and respiratory mechanics in women, as we discuss further [11].

### Sleep in post-menopausal age

Postmenopausal age is defined retrospectively after 12 consecutive months of amenorrhea. It represents the end of the reproductive period and ovarian failure with a decrease in estrogen levels. Despite intensive research, a correlation between polysomnographic features, menopausal stages, and hormonal levels is not clearly demonstrated. This may be due to the presence of multiple influencing factors, including a high variability of their measurements across the menopausal phase. In fact, polysomnographic data have shown either no differences or worse total sleep time, sleep fragmentation, and sleep efficiency in perimenopausal/postmenopausal women compared with younger women [12]. The worst period seems to be the perimenopause, when vasomotor symptoms, which occur in 70% of women, and hormonal fluctuations may cause fragmentation of sleep or awakenings. Psychosocial aspects can also affect sleep. In postmenopausal women, sleep quality is impaired by comorbidities such as mood changes, anxiety, depression and insomnia. Insomnia occurs in nearly 60% of postmenopausal women [13]. Although non-respiratory disorders are outside the scope of this review, insomnia is strongly associated with respiratory sleep disorders, particularly in women [14]. Insomnia is the most common sleep disorder in menopause compared to the prevalence of obstructive sleep apnoea (16-20%) or restless legs syndrome (20-24%), so a clinical evaluation or simple screening using the Insomnia Severity Index could be useful to detect our female patients. In recent years, the great knowledge about the coexistence of OSA and insomnia (COMISA) had increased the home sleep apnoea test or polysomnographic studies in women to detect also the presence of sleep disordered breathing [15, 16].

### Pathophysiology of obstructive sleep apnea in women

People have obstructive sleep apnea for different reasons. An anatomical factor is present in all patients (in 30% of patients it is the only factor) but, in 70% of cases, one or more non-anatomical factors coexist, which are responsible for a different endotype and phenotype of the disease [17].

The four pathophysiological factors that can contribute in a different way are: 1) pression critical of collapse (Pcrit) of the upper airway or anatomical factors (obesity, craniofacial conformations of reduced dimensions, laxity of the soft palate or macroglossia, etc.); 2) instability of ventilatory control, also known as high loop gain (LG); 3) neuromuscular inefficiency of the dilator muscles of the upper airways also known as poor muscle responsiveness; 4) increased propensity for nocturnal awakenings due to respiratory stimuli, or a reduced awakening threshold, also known as low arousal threshold (low AT). Gender differences in pathophysiology can translate into personalized approaches based on the specific endotype. Below we summarize what it is currently known on the pathophysiology of OSA in women.

#### Anatomical factors

Women have a smaller upper airway than men, which increases tracheal traction and reduces the likelihood of collapse [8]. Usually, obese women have a peripheral fat distribution that does not correlate with the fat distribution in the neck. Only severe obesity (BMI > 40) with a central distribution or some metabolic or hormonal diseases result in central obesity in premenopausal women. Furthermore, women have smaller lung volumes and diaphragm (reduced in length by about 9% and less strong compared to men), so REM sleep, the stage of sleep in which the diaphragm is responsible of almost all the work of breathing, is often the stage with more respiratory events in women. The severity of sleep-disordered breathing increases in menopause, but this increase is not related only to BMI or aging. Indeed, even if after menopause, women present a distribution of fat which is more similar to the central obesity of men the main anatomical change is in the upper airway that it increases in length and become more prone to collapse [18]. Nevertheless OSAS in non obese patients could be, also in women, caused by other anatomical factors [19]. A pathophysiological study of Jordan et al compared Pcrit in the two sexes, confirming that in men the collapsibility of the upper airway is greater than in their female counterparts, regardless of BMI and AHI [20].

#### Upper airway responsiveness

One of the most important protective factors to sleep apnea in women is the upper airway responsiveness. Progesterone increases the EMG activity of genioglossus muscle, the main airway dilatator muscle. This is well known from the 1998 when Popovic et al demonstrated that EMG activity of genioglossus increases in the luteal phase compared to the follicular one in line with progesterone levels and it dramatical decreases in the menopausal age promoting tongue collapse [21]. Other studies demonstrated that estrogen receptors have been identified in the pharyngeal muscles, experimental studies in animals have demonstrated that stimulation with estradiol accentuates the contractility of the genioglossal muscle and also hormonal therapy increases EMG activity of genioglossus muscle in menopausal women; however, this therapy did not give consistent results for treatment in menopause women with OSAS [22]. Hormone replacement therapy recommended by some clinicians during menopause cannot be used in all women due to its side effects. However, studies have shown that estrogen alone or estrogen-progesterone therapy provides a decrease in overall fat mass, improvement in AHI levels, improvement in insulin sensitivity and a decrease in the rate of development of type 2 diabetes [23, 24].

#### High loop gain

Knowledge about respiratory drive and control breathing in female are limited. Instable respiratory breathing or high loop gain (LG) is the most important but also the most complicated factor to understand sleep disorder breathing. Patients with a higher loop gain have an higher response to respiratory, which perpetuates respiratory events and results in unstable breathing [25]. This instability makes the upper airway more prone to collapse. Respiratory drive, which describes the output of respiratory centers to respiratory muscles, is different for each person and it is also modulated by hormonal changes [26]. Progesterone is a stimulator of respiratory drive. During pregnancy, progesterone levels increase, which compensates for the decrease in tidal volume related to the expanding abdomen by increasing the respiratory rate and respiratory alkalosis due to heightened respiratory drive activity. Nevertheless, it has been suggested that an excessive increase in respiratory drive results in an instable breathing and predispose to OSA even in pregnancy and more obese women [27].

Few pathophysiological studies have been made about this topic. Jordan et al demonstrated that the measure of respiratory control stability is not different between men and women with either equally severe OSA or equal BMI, nevertheless the authors themselves discuss about the power of this result to detect physiologically differences in LG [20]. Although other pathophysiological studies seem to support a non-different role of loop gain in the two sexes, some evidence seems to emphasize that high loop gain is clinically more relevant in the male sex [28, 29]. For example, periodic breathing, during either sleep or exercise, is not common in women [30]. On the other hand, it is necessary to take into account how the ventilatory drive and thus also the loop gain can change in women at different times of life: respiratory drive, in women decreases with the menopause. And it could be one of the reason because Obesity Hypoventilation Syndrome (OHS), in which there is a reduced response to ventilatory disturbance (low LG), is more frequent among obese menopausal women than in men [31]. Difference in LG in two sexes has been demonstrated in Central Sleep Apnea (CSA). A involvement in periodic breathing during sleep due to high altitude, in which women showed more stable breathing and appear protect to central sleep apnea related to altitude [32]. The prevalence of REM OSA in women, as described below, in contrast to the reduced frequency of NREM OSA and periodic breathing, both of which express high loop gain, further supports the idea that there may be a difference in this functional factor between the two sexes that has not yet been fully elucidated [33].

#### Low Arousal Threshold

Arousal Threshold (AT) is defined like the negative intra-esophageal pressure causing a cortical arousal. Indeed, patients end a respiratory event and reopen the airway when the negative intrathoracic pressure reaches the threshold of the negative intra-esophageal pressure that stimulates a mechanical or chemical reflex (threshold of recruitment) or if it reaches the pressure needed to give an arousal (arousal threshold). Patients with a low AT end the respiratory event because they reach the arousal threshold before reaching the threshold of recruitment [34]. For this reason in individual with low AT, when the arousal occurs the airway does not fully reopen, making it more susceptible to subsequent collapse. Cortical arousals and unstable sleep cause fluctuations in respiratory drive and CO2 levels and decrease time spent in N3 sleep, in which usually obstructive events are less common and breathing is more stable [35]. Women have a more instable sleep with a lower AT than men. Indeed, estrogens increase sleep stability, time spent in N3 sleep stage and decrease temperature and cortical arousal [36]. Nevertheless women are more susceptible to sleep disorder, like sleep fragmentation and insomnia than men [7]. During menopause, as estrogen levels decrease, sleep fragmentation increases, leading to secondary respiratory instability [7].

#### Pathophysiology beyond a schematic division

Beyond this schematic division, the physiological aspects are much more complex. Functional factors such as loop gain and arousal threshold are not static characteristics. They change throughout the night, vary across different sleep stages, and influence each other. Loop gain decreases from N1 to N3 and it also decreases in REM, while increasing during the night. Arousal threshold increases from N1 to N3 and during REM it is higher than in other sleep stages. For this reason, is not easy to identify one major functional factor in our patients, but sometimes it is possible to recognize some typical polysomnographic features related to one prevalent endotype. Low AT is common in women but their ventilatory response to arousal is reduced compared to that of men [37]. In fact, arousal only cause a slight post arousal ventilatory instability in women. This could be a factor contributing to the lower severity of the syndrome in women. After arousal, chemoreflex responsiveness returns to that of wakefulness. The ventilatory drive senses relative hypercapnia, inducing an increase in ventilation and a reduction in resistances. This explains the occurrence of post-arousal hyperventilation, which appears to differ between males and females [20]. According to Koo et al, this difference is due to the resistance of the airways in the two sexes [38]. Men have higher upper airway resistance, so after arousal, they hyperventilate more. This is not due to the CO2 drive but because of the sudden difference in airway resistance caused by the arousal. In addition, an increase in ventilation linked to arousal exists, which is called the waking reflex and is physiologically greater in men. In REM sleep, the respiratory drive changes, and respiratory regulation becomes behavioral rather than purely metabolic, with a dramatic decrease in EMG activity except for the diaphragm. This sleep stage is more problematic for women than for men for both mechanical and anatomical reasons. As described above, the diaphragm is shorter and weaker in women. The upper airway of women, compared to men, may be more prone to collapse in REM sleep due to its smaller size. Additionally, in REM sleep, when neuromuscular responsiveness is reduced, the respiratory drive is also diminished, further increasing the risk of airway collapse [39]. Therefore, even if women have a low arousal threshold (AT), they generally do not experience severe airway obstruction during NREM sleep compared to men. Instead, they often encounter flow limitations, respiratory arousals, or brief hypopneas, which may not lead to significant drops in oxygen saturation. During REM, especially obese women may have long obstructive events associated with severe desaturations [40].

In fact, despite low AT, the response to the change in blood gases in women during REM sleep stage is slower, therefore longer events with severe desaturations can occur. OSAS can occur in younger women during REM sleep, as the protective hormonal effects on genioglossus muscle activity are diminished in this sleep stage [41]. However, there is limited literature providing detailed data on the varying polysomnographic features or pathophysiological mechanisms of OSA across different ages in women. It is well know that OSA is an uncommon disease for women in premenopausal aging in absence of important risk factors like severe obesity or hormonal disfunction (e.g. polycystic ovarian syndrome) [11, 42]. This difference does not seem to be explained by differences in respiratory drive. Even a more central distribution of fat after menopause may not be the only factor justifying the different prevalence between pre- and post-menopausal women. Koo demonstrated that with menopause or aging, there is an increase in retropalatal collapsibility, and the sites of airway collapse become more numerous. Additionally, a reduced responsiveness of the airways may contribute to the fact that apneas are no longer restricted exclusively to the REM phase [43]. In addition, there is some plasticity in the muscles of the upper airways, suggesting that intermittent hypoxia stimulates the muscles’ ability to contract. However, this plasticity is lost after menopause. The main pathophysiological changes leading to the increased prevalence of OSA during menopause are complex and not yet fully understood, involving both hormonal and respiratory mechanics changes [44]. In literature some comorbidities increase the risk of OSA in woman patients. Chronic obstructive respiratory diseases and neuromuscular disorders are the main respiratory disorders associated with OSA. In particular, females have a higher likelihood of developing asthma and developing a more severe form of asthma than their male counterparts even if there is a big differences in prevalence in different age or hormonal status [45]. Severe asthma with associated frequent awakenings and obesity and also gastrointestinal reflux could increase risk of development OSA and also asthma increase the risk of non controlled asthma [46]. Literature and also our previous work demonstrated that Low AT is a common pathophysiological trait in OSA patients with Asthma, as well as in OSA female patients as we discuss earlier [47]. Among neuromuscular disorders with a respiratory involvement, Multiple Sclerosis is the most frequent in woman and it is associated with an increase risk of obstructive sleep apnea, in particular when women are obese or during the pregnancy [48, 49]. Another question in literature is that menopause itself is a risk factor for sleep disorder breathing. If menopause per se is responsible for OSA or the increase in sleep disorder breathing among post-menopausal women is due to the aging process and/or alterations in fat distribution related to menopause or also sleep disturbances menopause related, in literature, is yet questionable [50].

### Clinical presentation in women with OSAS

Female with OSAS report more monthly fluctuations in self-reported sleep quality, such as more sleep disturbances, including insomnia, frequent awakenings, non-restorative sleep, unpleasant dreams or nightmares. Symptoms about bad sleep are usually reported during the premenstrual week and the first days of menstruation compared to other menstrual phases [51]. The most important symptoms of OSA in women are related to both sleep problems and mood disorders like depression or anxiety, but also to cognitive impairment (e.g. loss of memory or attention). The typical symptoms of OSAS, like snoring, reported apnea, nocturia or sleepiness are not absent even if less reported [52]. In particular, snoring is poorly reported, because it is “unladylike,” but it is frequent in women after 50 years of age and it remains the most predictive symptom in both sex [42]. Women with OSA may report their sleepiness differently, often emphasizing a lack of energy, fatigue, or tiredness rather than typical sleepiness. As a result, they may score lower on the Epworth Sleepiness Scale, leading to an underestimation of this symptom [53]. Nocturia, defined as waking up to urinate two or more times per night, can be caused by the increased negative intrathoracic pressure during an apnea, hypopnea, or even a respiratory effort related arousal. This increased pressure leads to a secondary increase in venous return to the right side of the heart, causing myocardium stretch and the release of Atrial Natriuretic Peptide (ANP). Increased levels of ANP lead to natriuresis and urine production [54]. Nocturia is also specific, and it is more frequent among women than men (60% *vs* 40.9%, respectively). Despite being a specific symptom, nocturia is still not included in any screening questionnaires [55] (Table 1).

**Table 1:** Main symptoms of OSAS in women

Symptoms presented by women with OSAS
• Unrefreshing sleep
• Morning headaches
• Nocturia
• Snoring
• Insomnia
• Nightmares
• Concentration and memory impairment
• Mood changes
• Irritability
• Depression and Anxiety
• Sleep fragmentation and frequent awakenings

Differences in clinical and risk factors in women could also explain the underestimated prevalence of OSAS in female patients. In fact, the items on screening questionnaires used in the general population, like Berlin Questionnaire, STOP BANG etc, are more specific to men than women. The sensitivity and specificity of the various OSA screening questionnaires have been questioned in the literature. To date the best questionnaire for both sexes remains the Berlin questionnaire [56]. Only BMI, neck circumference, and a high Epworth Sleepiness Score were independently predictive of moderate to severe OSA in men, whereas age, neck circumference, and morning headache were independently predictive in women [57]. Other studies have shown that anthropometric variables, such as neck and abdominal circumference, are more likely to be associated with severe OSA in men [58]. In the STOP BANG questionnaire, the only item that is also positive for women is the BMI [59]. Screening questionnaires available to date are predictive in severe or moderate cases, while women with OSAS have symptoms and a poorer quality of life even in mild cases that are not been detected by this questionnaire. In women, the presence of a respiratory sleep disorder seems to be more related to changes in sleep quality and quantity. Summarize the symptoms related to sleep disturbance and mood changes are difficult to detect by common screening methods [53].

### Polysomnographic features

Concerning the polysomnographic features of OSA, four major differences have been reported between the two sexes [60]. First, women with OSA have more awakenings, and a worse sleep quality. Second, they have milder OSA, with more hypopneas or flow limitation than apneas. Third, women with OSA have a greater proportion of their respiratory events during REM sleep [61]. Four there is a prevalence of events detected only with the polysomnographic study with EEG as flow limitation arousal or RERA. These differences between the two sexes do not appear to be age- or weight related, but one possible explanation could be the prevalence of low AT in female even if few pathophysiological studies exist [5, 62]. In the clinical setting, women with OSA present two main polysomnographic patterns.

**Fig. 1:**
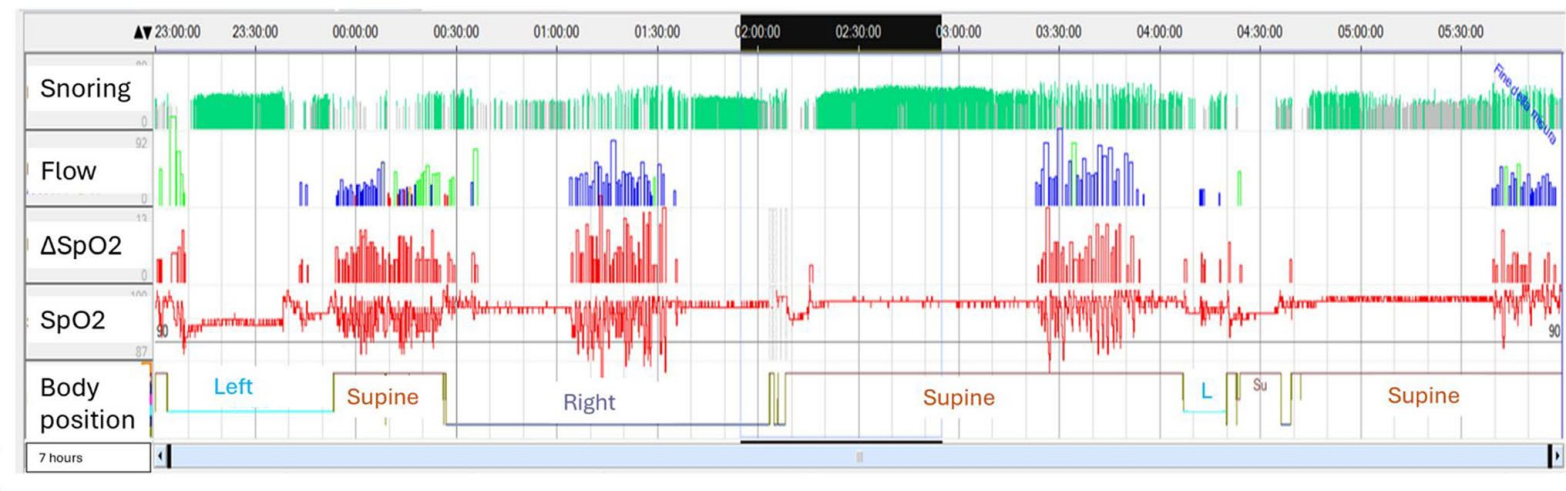
Recording of the whole night of a middle age women with OSA that she complain depression and anxiety. This a home sleep apnea test but the cluster of respiratory events suggest a typical pattern of REM OSA

The first one is OSA REM (Fig. 1). The definition of OSA REM varies in the literature, but one of the most used definition is as twice as many respiratory events in REM sleep compared to non-REM sleep [63]. Women have more frequently OSA REM, while men have more frequent positional OSA [63]. OSA REM is more common in the first 55 years, but some studies suggest that in women it is more common in older women [64]. These difference is most likely due to the different definition of OSA REM adopted. Nevertheless, women OSA REM tend to be more obese and affected by depression [65]. REM-related SDB has been linked to an increased risk of incident hypertension, metabolic syndrome, and diabetes [66, 67].

**Fig. 2:**
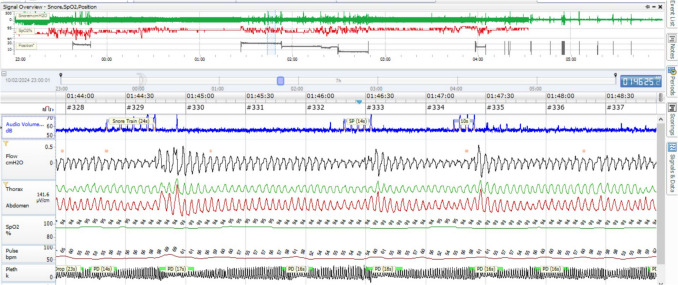
Home sleep apnea test in a female 45 years old OSA patient with insomnia. Upper part: the recording of whole night. Patient with Low AT. AHI < 10. Bottom part: 5 min of sleep recording. There are flow limitation events followed by autonomic activation with resumption of ventilation, but without a desaturation. These events are possible expression of flow limitation arousal

The second typical polysomnographic pattern in women is the presence of flow limitation with cortical arousal, also known as RERA (Fig. 2) [68]. With the use of home sleep apnea tests, female patients are often underestimated in terms of sleep apnea severity. However, it is possible to recognize or suspect flow limitation arousals in home sleep apnea tests without EEG. This can be done by observing pulse wave drops or significant breaths following events, which can be identified by an experienced clinician. OSA female patients usually have short events, shallow desaturations (with the exception of the REM sleep stage), hypopnea, and flow limitation arousal: all these are features of low arousal threshold [69]. Cortical arousal can terminate an apnea before a deeper desaturation occurs, leading to a lower apnea-hypopnea index (AHI) despite significant cortical and sympathetic activation. In these patients, increased upper airway resistance can heighten the work of breathing, causing arousals, disrupted sleep, and impaired daytime cognitive function. Upper airway resistance alone, without complete obstructive apnea or respiratory disturbances, has been shown to produce clinical symptoms such as daytime fatigue and depression, both of which are reported by women with OSA. Some authors suggest the importance to treat the female patients with this polysomnographic pattern, even if their AHI is less than 5, because these patients are more symptomatic and exhibit significant fragmented sleep [70].

The two previously described polysomnographic patterns, often coexist. Indeed, this indicates low AT as principal functional factor [71, 72]. Nevertheless, after menopause, with aging, obesity, or other comorbidities, polysomnographic patterns in women could change and become more similar to those observed in men (Table 2).

**Table 2:** Main polysomnographic characteristics in women with OSAS

Polysomnographic characteristics in women	
• Sleep time: longer but fragmented, longer sleep latency, reduced N3 sleep stages, more awakenings, worse sleep quality	
• Minor AHI	
• Prevalence of hypopneas, flow limitation, RERA, short events	
• REM OSA with longer events (hypopnea)	
• Positional OSA less frequent	
• Coexistence of periodic breathing less frequent	

### OSAS treatment in women

CPAP treatment in obstructive sleep apnea is always effective, as CPAP reopens the airways independently of the site of collapse. Different adherences to CPAP between two sexes have been reported in the literature [73, 74]. This may be due to the fact that women have a more negative critical pressure, meaning the pressure required to reopen the airway is lower than in men. However, as previously discussed, symptoms in women can be related to respiratory events but also only to flow limitations or arousal from sleep. For these reasons, correcting flow limitations and snoring is probably as important as addressing hypopneas and apneas in women. These factors make women often more complicated to treat with a phenotype-targeting approach [17, 75]. Women with OSA often complain about insomnia, leading to the development of a syndrome called COMISA (co- occurring insomnia and sleep apnea) [15, 34, 76]. When choosing the treatment for female patients with OSA, it is important to first ask about insomnia. In fact, if insomnia is present, a pharmacological or cognitive behavioral treatment (CBT-I) should precede CPAP or other therapies for sleep apnea [77]. The treatment of choice of insomnia, with or without OSA, is cognitive behavioural therapy. As a second line of treatment, melatonin, benzodiazepines and Z-drugs could be useful. Some studies have shown that hormone therapy can improve sleep quality. However, a recent meta-analysis reported an improvement in quality of life with hormo therapy only in patients with premenopausal vasomotor dysfunction [2, 16]. Pharmacological treatments for insomnia are widely used in practice. Nearly 50% and 90% of insomnia patients, in the USA and Japan respectively, choose pharmacotherapy [78]. The most commonly used drugs for insomnia are benzodiazepine receptor agonists, including GABA modulators like benzodiazepines (BDZs) and benzodiazepine-like Z-drugs. BDZs are effective short-term but carry risks such as dependence and next-day hangover. Z-drugs (zolpidem, zaleplon, and eszopiclone) became popular after the 1980 s for their lower abuse risk. They selectively act on GABA receptors and enhance GABA-mediated inhibition, reducing the time to fall asleep and improving sleep quality [79]. When OSA and insomnia coexist like for example in patients with COMISA, Zolpidem, alone or with CPAP, could be useful for his “paradox effect” to increase muscle upper airway contraction [80]. Dual orexin receptor antagonists (DORAs), have been approved for treatment of insomnia. Orexin is a neuropeptide that plays a role in the promotion of wakefulness, The DORAs act by targeting the orexin cascade through the antagonism of both receptors, promoting sleep induction and maintenance, with a more localized effect [81].

DORAs were the best recommendation in sleep maintenance insomnia with greater efficacy and tolerability. Regarding the safety profile of DORAs, a recent metanalysis has been showed mild adverse effect: somnolence, nasopharyngitis, and headache [82]. On the other hand, women with low AT may benefit from pharmacological treatment, with or without CPAP treatment, like zolpidem, eszoplicone or other hypnotics [83]. Titration CPAP in women requires precautions related to the characteristics of sleep and disease in female sex. A first approach usually involves using a lower pressure and a time to reach target pressure (i.e. ramp) longer than commonly used in men, paying attention to residual snoring and flow limitations. Some auto CPAP machines have specific algorithms for the breathing profile of women with OSA, which can recognize and correct flow limitation [64]. Despite the fact that menopause is a risk factor for OSA, hormone replacement therapy has not been shown to be effective in the treatment of sleep disordered breathing [50].

### OSA treatment when Restless Legs Syndrome or Periodic Limb Movement coexist

Restless Legs Syndrome (RLS) and Periodic Limb Movement (PLM) are both common in female patients and in patients with OSA. RLS is characterized by an urge to move the legs or arms, commonly with uncomfortable dysesthesia, with the difficulties initiation and/or maintaining sleep. RLS is classified as primary or secondary in origin (iron deficiency, end-stage renal disease, or pregnancy) [84]. The higher prevalence in women may be associated to fluctuation in dopamine levels [85, 86]. OSA and RLS, which are prevalent in the general population, often occur together in the same patient, leading to an increase in the severity of both conditions. RLS may cause sleep fragmentation even with appropriate treatment for sleep-disordered breathing or complicate CPAP therapy. Treating RLS with sleep-disordered breathing should begin with conservative measures and addressing any underlying causes (avoidance of substances or medications that may exacerbate RLS, and oral and intravenous iron supplementation etc). If RLS persists or affects sleep quality and life and CPAP adherence, pharmacological treatment (with alpha-2-delta ligand calcium channel blockers, gabapentin, pregabalin and gabapentin enacarbil) should be considered [87]. PLM are detected on polysomnography as brief (0.5–10 seconds), recurrent flexion movements of the lower extremities (most common) that occur roughly every 15–30 seconds [88]. Coexistence is estimated at 24-28% [89]. Some trials suggest CPAP can reduce PLMS severity in OSA patients [90].

Severe OSA can hide PLM, which may worsen after CPAP treatment. In mild or moderate OSA, PLMs may be triggered by respiratory arousals but improve with CPAP. Thus, CPAP could be the first step to treat obstructive apnoea, though it may not fully resolve periodic limb movements. Persistent PLM in OSA patients could cause poor sleep quality and daytime sleepiness, reducing CPAP compliance. It’s best to detect PLM at diagnosis and inform patients about potential sleep deterioration to prevent CPAP discontinuation. A meta-analysis suggests clinicians should monitor PLM in patients undergoing CPAP therapy for OSA with a full polysomnography or an home sleep apnea test with legs electrodes [91]. PLM is often linked with RLS, and dopaminergic medications like pramipexole, ropinirole, and rotigotine are used to manage these conditions, which can reduce PLM and improve sleep quality [88]. Ongoing research continues to enhance our understanding and management of these conditions.

### Pregnancy and OSA

Pregnancy involves anatomical and functional changes that increase a woman’s risk of obstructive sleep apnea. Despite elevated progesterone levels enhancing genioglossus muscle activity and boosting respiratory drive—thereby increasing ventilation even when tidal volume is reduced in some susceptible individuals—this is often insufficient to prevent apneas [92].

First, anatomically, pregnant women have an increase in abdominal volume due to the growing uterus, which raises the diaphragm and reduces its contractility. This is particularly problematic during REM sleep, when the diaphragm is the primary respiratory muscle, causing a reduced tracheal traction on the pharynx makes it more prone to collapse. However, it is important to note that REM sleep is generally reduced during pregnancy [27].

A second anatomical factor is edema of the upper airway tissues, which is associated with increased progesterone levels. This edema can promote airway collapse and, in pregnant women, may lead to increased oral breathing and resistance in the upper airways, further contributing to their collapse. Additionally, while increased respiratory drive is generally beneficial, if it becomes excessive, it can lead to respiratory instability and a higher susceptibility to respiratory events [93]. Sleep fragmentation and cortical arousal as well as the presence of sleep stages instability and secondary fluctuations in CO2 and respiratory drive predispose to obstructive respiratory events. All these predisposing factors may be exacerbated in the presence of obesity or in conditions such as asthma and multiple sclerosis, which are more common in women of childbearing age.

Recent studies have shown that the presence of OSA in pregnancy is associated with hypertension, gestational diabetes, pre-eclampsia and major pre- and peripartum complications [94]. Furthermore, OSA in pregnant women is associated with preterm birth of the baby, low Apgar index and low birth weight, even of evidence is more sparse [95]. There is insufficient data in the literature to show that CPAP treatment of high-risk women with OSA improves metabolic and hypertensive profiles and reduces fetal complications, due to the small number of clinical trials in the literature [96].

Despite recent anesthesiologic recommendations that focus attention on this risk category, obstetrics guidelines do not recommend screening for OSA in pregnant women, but only in women with OSA and BMI> 30, hypertension or complications such as diabetes or hypertension in a previous pregnancy [97]. To note, screening questionnaires are not sufficiently sensitive in pregnant women [98].

Given these considerations, it is clear that the presence of OSA during pregnancy necessitates thorough anamnesis and the implementation of screening for the entire pregnant population, as well as cardiorespiratory monitoring for those at higher risk.

### Polycystic ovarian syndrome and OSAS

The only pathological condition that increases the risk of OSAS in women of childbearing age is the polycystic ovary syndrome (PCOS). Indeed, reduced progesterone levels and hyperandrogenism promote airway collapsibility and alter the ventilatory response of the chemoreceptors. In PCOS, women lack the protective hormonal effects on upper airway responsiveness. Obesity is often central, accompanied by systemic inflammation, and reduced estrogen levels contribute to further disturbances in sleep stability [11]. However, the two diseases influence each other: the effect of hyperandrogenism on the risk of OSA in women with PCOS is likely to be mild, as androgen levels are low compared to men, but sleep quality seems to play an important role in female hormone production. In fact, sleep deprivation, sleep fragmentation and the presence of respiratory events are thought to affect the release of gonadotropin-releasing hormone (GnRH), follicle-stimulating hormone (FSH) and luteinizing hormone (LH), with subsequent changes in the menstrual and ovarian cycles [99]. Consequently, OSA may alter sex hormone production and contribute to the development or worsening of PCOS [100, 101].

## Conclusion

Women with OSA are frequently misdiagnosed or underdiagnosed due to a reluctance to acknowledge symptoms and seek medical help, highlighting gender differences in diagnosis. Historically, clinical trials and research focused primarily on OSA in men, so the specific clinical and polysomnographic features relevant to women were not fully considered until recently. OSA in women is a significant concern: untreated OSA is associated with a higher incidence of stroke, chronic heart disease, and poorer quality of life. Special attention is needed for women during menopause, pregnancy, and those with PCOS in sleep respiratory medicine. Addressing gender differences in sleep respiratory medicine is a crucial step toward personalized care.

## Data Availability

My manuscript has no associated data.

## Abbreviations

*AHI *: Apnea Hyponea Index
*ANP*: Atrium Natriuretic Peptide
*BMI *: Body Mass Index
*CPAP *: Continuous Positive Air Pressure
*COMISA *: Co-occurring insomnia and sleep apnea
*EMG *: Electromyographic
*FSH *: Follicle-stimulating hormone
*GnRH *: Gonadotropin-releasing hormone
*LG *: Loop Gain
*LH *: Luteinizing Hormone
*Low AT *: Low Arousal Threshold
*NREM *: Non Rapid Eye Movements
*OHS *: Obesity Hypoventilation Syndrome
*OSAS *: Obstructive Sleep Apnea Syndrome
*PCOS *: Poly Cystic Ovary Syndrome
*REM *: Rapid Eye Movements
*SDB *: Sleep Disordered Breathing
